# Inverse design of compact power divider with arbitrary outputs for 5G applications

**DOI:** 10.1038/s41598-022-17212-0

**Published:** 2022-07-27

**Authors:** Maryam Shadi, Mohammad Reza Tavakol, Zahra Atlasbaf

**Affiliations:** 1 Department of Electrical and Computer Engineering, Tarbiat Modares University, Tehran, 14115 Iran; 2 Electrical Engineering Department, Sharif University of Technology, Tehran, 11155-4363 Iran

**Keywords:** Engineering, Electrical and electronic engineering

## Abstract

Since the recent on-demand applications need more sophisticated circuits and subsystems, components with configurable capabilities attract attention more than before in commercial systems, specifically the fifth generation (5G). Power dividers play a crucial role in 5G phased array systems, and their role becomes more significant if the output powers ratio is adjustable. Here, we suggest a design methodology by which planar power splitters with arbitrary output power levels can be designed in light of very simple perturbations, i.e., vias. Through our design procedure, we find an optimized pattern for hybrid vias-some of them are made of PEC, and others are dielectric, e.g., air, high-permittivity materials. Thanks to deep neural networks, we demonstrate that this technique can be employed to design power splitters whose output ports have different amplitudes. In light of the proposed method, we fabricated and measured a 4-way power divider realizing Chebyshev coefficients for sidelobe reduction of a 4-element array at 28 GHz as a proof-of-concept. We believe that this methodology in which hybrid perturbation is the key spot paves a way to implement complex functions in various platforms and other structures, e.g., SIWs, ridge waveguides, rather than the one we investigated (planar/microstrip).

## Introduction

Wireless communication technology has advanced dramatically in the last decade by novel techniques of controlling electromagnetic waves transmission, propagation, and detection more effectively, e.g. reconfigurable radiators and metasurfaces^1–5^. Accordingly, different generations of communication networks have been suggested, among which the fifth-generation (5G) cellular networks attract more attention, specifically in terms of demand and practice. The 5G cellular network technology offers better data throughput, more energy efficiency, and reduced latency in light of 5G hardware development and transceivers’ gain increase^6,7^. In this way, an antenna system should be directive and at the same time have a low sidelobe level to realize a sharp pencil beam. Phased array antennas are a prominent configuration to achieve sharp beams in 5G systems^6,8^. A minimum number of RF chains, e.g., variable gain units and phase shifters, should be considered in phased arrays architecture^9–12^.

In array or subarray configurations, one of the essential modules is the power divider. Apart from power dividing, it is required to control the signal’s amplitude for some specific applications, such as sidelobe level reduction^11,13^. To now, different power splitting networks whose basis units are Wilkinson (or other typical types) power dividers have been introduced, but this type of divider is not capable of providing arbitrary output power ratio without additional gain/attenuation control units^14–19^. Also, other types of power splitting networks based on SIW structures cannot provide such functionality without different and distinct tunable elements^15,20,21^. Recently, photonic devices have been developed to control the power in the outputs with different ratios. The ratio can be reset by artificially engineering the device structure, e.g., adding geometrical or material perturbations into the guided region, by which electromagnetic field distributions or localizations are tailored^22^. It should be noted that not only has reconfigurable power splitting been realized, but also other functionalities like mode converting and multiplexing have been suggested^23^. To model and design complex functions, researchers tend to run bunches of full-wave simulations, and this is not affordable for structures with many details. Additionally, when these simulations are connected with the optimization process to find the target device/function, computational resource usage becomes higher and higher and thus more costly. Accordingly, newly recent works employed deep learning approaches and neural networks to predict sophisticated input-output relationships with better performance in terms of time and computations^24,25^.

In this paper, we propose a design methodology to develop compact power splitting circuits with the capability of defining arbitrary outputs and division ratios. This design methodology is based on subwavelength hybrid perturbations, i.e., two types of inclusions are added to the structure rather than only one type. We have chosen PEC vias as the first type and dielectric vias as the second type. These two types and their combination provide a versatile mechanism for electromagnetic field modification and localization in the device.

Afterward, we employ Neural Networks for optimizing the pattern of vias for each functionality, e.g., different power division ratios at the device’s outputs. To demonstrate our idea in practice, we designed and fabricated a 4-output power dividing module at 28 GHz based on Chebyshev coefficients, which can be operated as an amplitude controller in a phased array’s feeding network.

**Figure 1:**
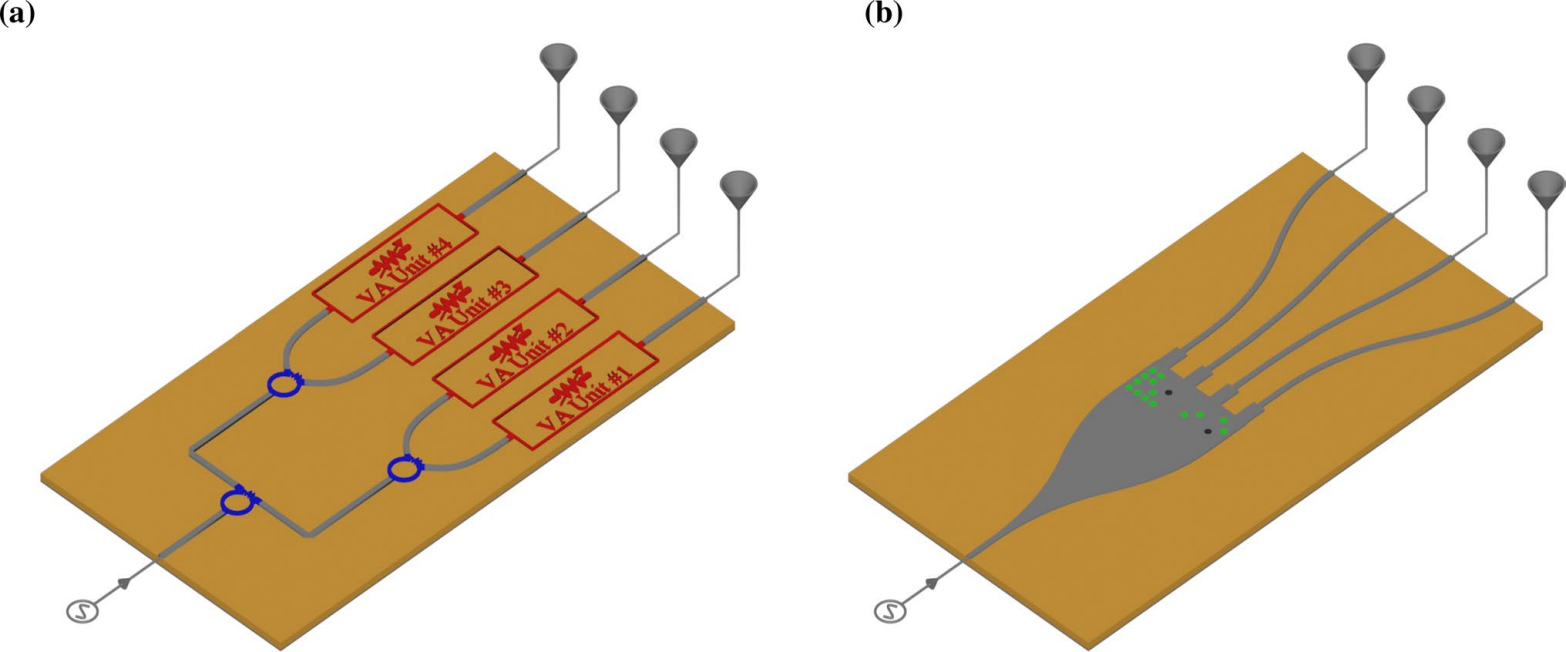
(**a**) Traditional feeding network in sub-array antenna (**b**) new compact and affordable structure to control amplitude level of primary array’s elements.

**Figure 2:**
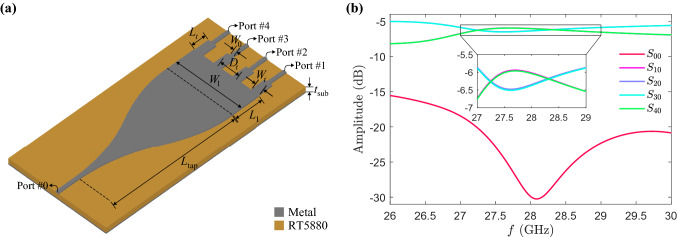
(**a**) Schematic of the proposed power splitter, not including any vias, for which the geometry parameters are $L_\mathrm{{tap}}=15$, $L_1=3.8$, $W_1=8$, $L_t=1.68$, $W_t=0.97$, $D_t=2.3$, $W_0=0.39$ and $t_\mathrm{{sub}}=0.127$ all in the unit of mm. (**b**) Transmission coefficients from the input port (No. 0) to the output ports (Nos. 1, 2, 3, 4), as well as input reflection coefficient, as functions of frequency.

## Proposed structure

For a wide variety of wireless communications applications, such as 5G technology, it is required to develop blocks or modules controlling the signal amplitude in separate channels. In this way, designers tend to use individual units, e.g., Integrated Circuits (ICs), to manipulate signals in each channel. This common point of view makes modules both extended and complicated. For example, consider a 4-channel transmitter feeding an antenna array, and we are supposed to manipulate the input power for each antenna to decrease the side-lobe level (SLL). To complete such a system, ordinary designers may use distinct attenuation or gain units and traditional power dividing schemes, e.g., Wilkinson power divider, see Fig. 1a. Conversely, as shown in Fig. 1b, this device can be realized in a monolithic and compact manner using to get the same functionality. Such architecture, together with inverse design methods, grants us the capability of providing different power ratios for the channels.

**Figure 3:**
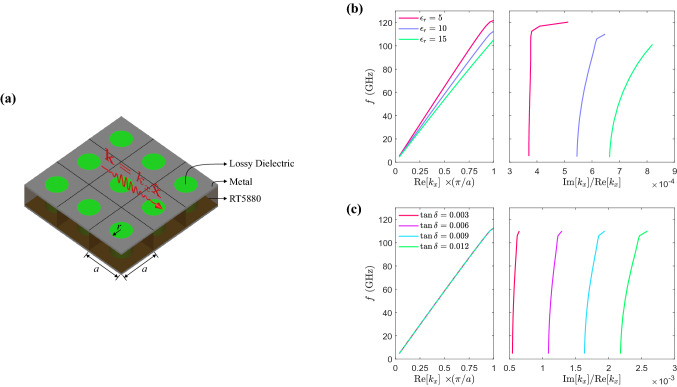
(**a**) periodic array of dielectric vias embedded in the grounded substrate, of which the upper side is covered by metal in areas without holes. The geometric parameters of the structure are $a = 0.7$ mm and $r =0.2$ mm. (**b**) dispersion curves for the mode propagating along the red arrow, i.e., the *x*-direction, in the periodic structure (**a**) for some discrete value of $\varepsilon _r$. (**c**) dispersion curves for variation of loss tangents for $\varepsilon _r = 10$.

**Figure 4:**
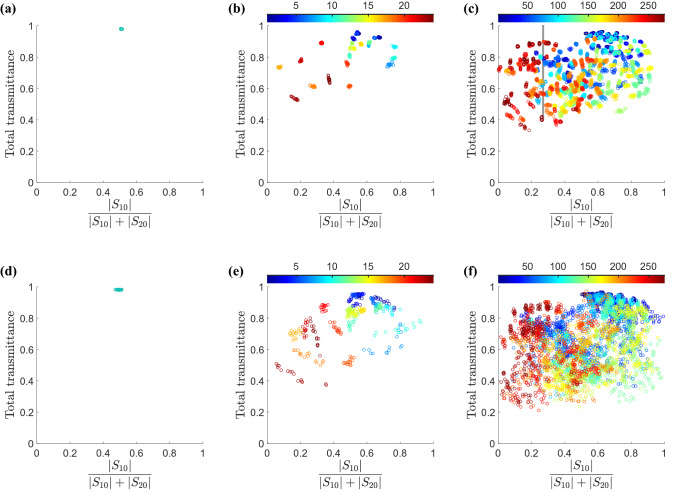
Total transmittance of symmetrical power divider versus division ratio for different vias distribution. Randomly vias patterns are considered for three combinations of hybrid PEC/air vias patterns shown in the first three figures: (**a**) only air vias without any PEC vias and 360 bunch simulations for the position of air vias. (**b**) air vias pattern combination with only one PEC via. The PEC via is located in 24 (C(24, 1) = 24 ) possible positions depicted by different colors. (**c**) air vias pattern combination with two PEC vias. The two PEC vias are located in 276 (C(24,2) = 276) possible positions depicted by different colors. Also, the three modes of hybrid PEC/dielectric vias patterns are in the second row of the figure. (**d**) 360 bunch simulations for dielectric vias pattern without any PEC vias. (**e**) dielectric vias pattern combination with only one PEC via. (**f**) dielectric vias pattern combination with two PEC via. For each position of PEC vias, consider 15 bunches of air vias position that show in the group by similar color.

To design the structure, making different power ratios at its outputs, we should introduce the initial platform by which this capability is attainable. We select and develop microstrip circuit elements since not only are they practical and customary, but also they have an acceptable performance within our operating band, i.e., from 27.5 to 28.5 GHz, in terms of loss. To begin with, the architecture upon which our proposed device is developed as shown in Fig. 2a. The illustrated device in this figure has been chosen as the perturbation-free structure (without any vias) that we will add vias to it in our process. In the proposed planar structure, as shown in Fig. 2a, the power dividing process starts with the tapered region, where the fields’ profile is being expanded.The tapered region is found by bezier curved^26^, which connects the input line to the wider multimode region very smoothly. Then four output lines are connected at the end of the wide untapered section, and transformers are mediated between the output lines with the impedance of $Z_0 =50\ \Omega$ and the wide end with the impedance of $Z_1=3.82\ \Omega$ for impedance matching. The four identical output branches are assumed to be parallel to each other that each microstrip line is equivalent to the quarter of expander admittance, so the quarter-wave transformers’ impedance is simply calculated by $Z_t = \sqrt{4Z_0 Z_1}$. Subsequently, the obtained values for the transformer width and length ($W_t$ and $L_t$) are set as the initial values, and by a light optimization process, we obtain $W_t$, $L_t$, and the output branches spacing ($D_t$). The device’s substrate is RT/duroid 5880 with a thickness of 0.127 mm, for which the loss tangent is assumed to be 0.0001. For this device, scattering parameters in dB are plotted in Fig. 2b. At the center frequency, the return loss ($\left| {S_{00}}\right|$) is as good as 30 dB, and the transmission to the output ports ($\left| {S_{10}}\right|$, $\left| {S_{20}}\right|$, $\left| {S_{30}}\right|$, and $\left| {S_{40}}\right|$) are almost equal, and their differences are less than 0.5 dB in 2 GHz bandwidth.

As we mentioned before, the microstrip structure’s guided-mode (wave) is supposed to be manipulated locally to provide various power ratios at the output ports. In this way, we chose vias made of a dielectric/air to control the local field amplitudes based on reducing reflections and scatterings. The effect of vias made of material with a higher permittivity on the guided mode can be analyzed using the dispersion associated with a two-dimensional periodic array of vias embedded in the substrate (as shown in Fig. 3a), and then we can understand the behavior of the effective (guided) medium they provide. In Fig. 3b, we demonstrate how dielectric vias with varying of $\varepsilon _r$ of 5, 10, and 15 and the identical loss tangents ($\sim 0.003$) can control the dispersion of guided mode in the structure. As can be observed, around the operating frequency, which is 28 GHz, the dispersion’s behavior is linear with the variable slope. In Fig. 3c, we investigate how dielectric vias with different loss tangent values affect the guided-mode propagation in the structure. As can be observed, the dispersion’s behavior is linear around the operating frequency, and the ratio between the attenuation and phase constants approximately ranges from 0.0005 to 0.0025 for loss tangent values of 0.003, 0.006, 0.009, and 0.0012.

In terms of the proposed construction, it is obvious that the materials utilized must have the potential to fill vias location.As an example, we used material with a dielectric constant of 10 and a loss tangent in the range of 0.003, which are routine candidates as 3D-printing materials for 5G applications^27,28^.

Consider a device whose structure is the same as the one shown in Fig. 2a, and vias, as perturbations, are added to this device symmetrically. Then the transmitted power to the inner ports is the same, i.e., $S_{10}=S_{40}$, and likewise, for the outer ports, i.e., $S_{20}=S_{30}$. The added vias will be distributed in a 4$\times$11 arrangement in the multi-mode (expanded) region. The spacing and other geometrical parameters for each element are the same as the ones for the unit cell in Fig. 3a.

Although the dielectric/air vias provide more material contrast than without any vias, the induced fields modifications by permittivity differences do not adequately alter the fields in the multi-mode region of the device. Therefore, we add finite PEC vias together with the dielectric/air vias to provide more perturbation for the fields. It should be noted that the number of PEC vias should be very few since more PEC vias modify the fields distribution substantially, and the resulted return loss and unwanted radiations deteriorate the device performance.To illustrate the effect of PEC vias, we have performed a bunch of full-wave simulations for the device, including a random dielectric/air vias pattern and one/two PEC via(s). On the other hand, we also have simulated the device, including only dielectric/air vias without any PEC vias. Fig. 4 indicates the total transmittance versus output signals levels ratio for different structures that may have no, one or two PEC vias combined with randomly distributed dielectric/air vias–we call each structure a sample.

**Figure 5:**
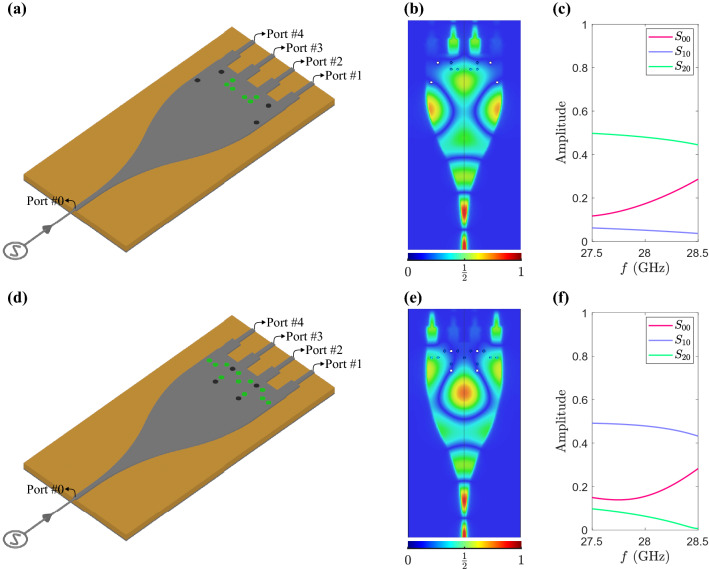
(**a**) and (**d**) Two different distributions for dielectric and PEC vias leading to transmittances which are totally dissimilar. (**b**) and (**e**) Normalized electric field profiles, which are full-wave simulation results. (**c**) and (**f**) Amplitude of scattering parameters for each via pattern.

The samples in Fig. 4a,d only have dielectric/air vias respectively , do not include any PEC vias, and their power ratio values concentrate around 0.5 ($S_{10}$/$S_{20}$ $\sim 1$), which may not be enough for multidisciplinary designs. Contrarily, for being not centralized power ratio values around 0.5, the diversity of samples of randomized dielectric/air vias with single and double PEC via are shown in Fig. 4b,c,e,f that are much more than the cases in Fig. 4b,e. A comparison between the result of these six simulations bunch verifies that a combination of dielectric/air vias and few PEC vias provide adequate fields perturbations, leading to diverse power ratio values, i.e., from 0.1 to 0.95 ($0.1<\left| {{S_{10}}/{S_{20}}} \right| <19$) . Also, those samples have acceptable performance in terms of the reflected and transmitted powers of the device. Consequently, we select a hybrid pattern, i.e., a randomly distributed dielectric/air and PEC vias, specifically two PEC vias, which can provide signal levels at output ports with arbitrary ratios. The desired power ratio determines the decision between dielectric and air vias; as shown in Fig. 5a , dielectric vias have a higher dispersion power ratio than air vias.

Here, it is worthy that we investigate two cases from dielectric hybrid vias whose power ratios are near to maximum and minimum values, and in Fig. 5a,d, the schematic of the device and vias pattern are displayed, and in Fig. 5b,e, the reflections and transmissions to the output ports are plotted within the frequency range from 27.5 to 28.5 GHz.

As evident from these plots, by choosing either vias pattern in Fig. 5a,c, we can swap the power ratio value 0.1 for 19, which are in extreme opposition to each other for the value of output ratio with the same return loss.Totally,we suggested a device with a wide range of output ratios, whose architecture can be selected depending on the application. Also, the electric field distributions in Fig. 5b,e verify the plots in Fig. 5c,f, respectively, i.e., for the case schematically shown in Fig. 5a,d, the electric field amplitude in the inner (outer) lines, namely ports 2 and 3 (1 and 4), is much higher than one in the outer (inner) lines.

We have $4 \times 6$ positions in the half of the device for the studied arrangement, being symmetric. In the selected hybrid scheme for this device, the total cases are $C(24,2) \times 2^{22}$ (*C* denotes combination) and can provide any values for the power ratio. The pattern associated with the desired case with defined scattering parameters can be determined by inverse design methods, e.g., Neural Networks (NNs), which will be explained in the following sections.

## Inverse design method to predict vias distributions

This section aims to clarify the inverse design procedure using NNs to find an optimal perturbation pattern, i.e., hybrid vias array, for our proposed architecture introduced in the previous section. Let’s define the problem and the inverse design method more precisely. It is assumed that we have desired spectra for the input reflection coefficient and the outputs transmission coefficients, and it is supposed to find a vias array so that the device response fits the target coefficients within the operating frequency range. Responses we investigated for the inverse network training are in the frequency range between 26 and 30 GHz, which is broader than the working frequency range since a wider frequency response covers more different mode resonant behaviors by which the device performance is defined more accurately. In this way, we choose samples within that range for each coefficient which is a function of frequency, i.e., $S_{00}$, $S_{10}$, $S_{20}$–total inputs are three times the samples number of each scattering parameter. The network’s output is a binary vector determining the presence of a via in each array cell.We employ NNs to solve this problem, which we call inverse networks.

**Figure 6:**
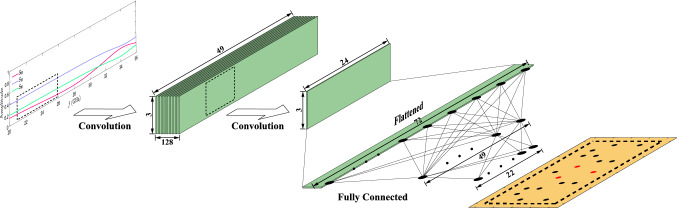
Overview of the DNN inverse design. The scattering parameters of the symmetrical structure are the input of the network. The first convolutional layer has 128 channels with a kernel size of 5$\times$3, and the second convolutional layer has one channel with a kernel size of 3$\times$3. The max-pooling stride of both the 2D-convolutional layers is (2,1). The output of the second convolutional layer is 24$\times$3, which is flattened to 72$\times$1 as the fully connected layers’ input.

As discussed previously, we consider the hybrid scheme for vias arrays of our symmetric device, where 2 PEC vias’ positions are fixed, and 22 other cells are for the dielectric/air vias, not being fixed. For each architecture determined by the fixed PEC vias, we assign an inverse network to solve a classification problem, leading to finding the randomly distributed dielectric vias pattern on those 22 positions. As a result, the network output is defined by a 22-element binary matrix covering $2^{22}$ total cases, or so-called labels. We provide 101 samples (totally 303) for each scattering parameter for the network as the input. To train such a network for inverse modeling, we need huge training data, which should be provided by full-wave simulations.

**Figure 7:**
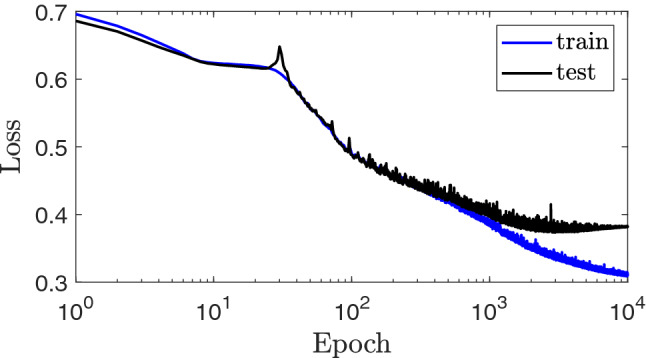
Behavioral of training network curve for both training (blue line) and test (black line) losses for $\sim 10,000$ epochs, based on the Binary Cross-Entropy as cost functions, which has a low loss value of $\sim 0.37$.

The inverse network structure is composed of two fully connected layers with the Sigmoid and ReLU activation functions, followed by two 2D deconvolution layers, as illustrated in Fig. 6.The NN is trained to forecast dielectric vias to minimize binary cross-entropy (BCE) loss, As follows^29^:

1 $$\begin{aligned} {\mathrm {BCE}}=-\sum _{i= 1} ^m \left[ y_i\log \hat{x_i}+(1-y_i)\log (1-\hat{x_i})\right] \end{aligned}$$

where $y_i$ represents the $i_{th}$ value of the training data obtained by full-wave simulation, $x_i$ is the output of DNN based on $y_i$ estimation, and m denotes the maximum number of via’s positions. The prediction value of each via’s location can take any weight between 0 and 1, which is quantized to any number of zero or one according to the threshold defined (we consumed 0.5). A total of 1500 samples are generated by full-wave simulation without any duplicated patterns used as training data. We use PyTorch, an open-source machine learning framework written in Python, to build and test our inverse networks on a high-performance computer with a CPU board.The loss function for the representative network parameters, as shown in Fig. 7 with batch size of 128, epoch number of 10,000, and trained on 1500 data is better than of $\sim 0.37$. We analyze the design performance on arbitrary scenarios to assess the network’s capabilities.

We try to optimize the device for the third-order Chebyshev coefficient at a goal splitting ratio of 0.37 ($S_{10}$/$S_{20}$), which is the same as the SLL of 40 dB.The candidate typologies for our required design are shown in Fig. 4c with the vertical gray bar, and we chose the topology with a total transmittance of 0.91 to achieve the desired insertion loss. DNN outputs a binary matrix, which is subsequently fed back into the full-wave solver, and the simulation result is shown in Fig. 9.

**Figure 8:**
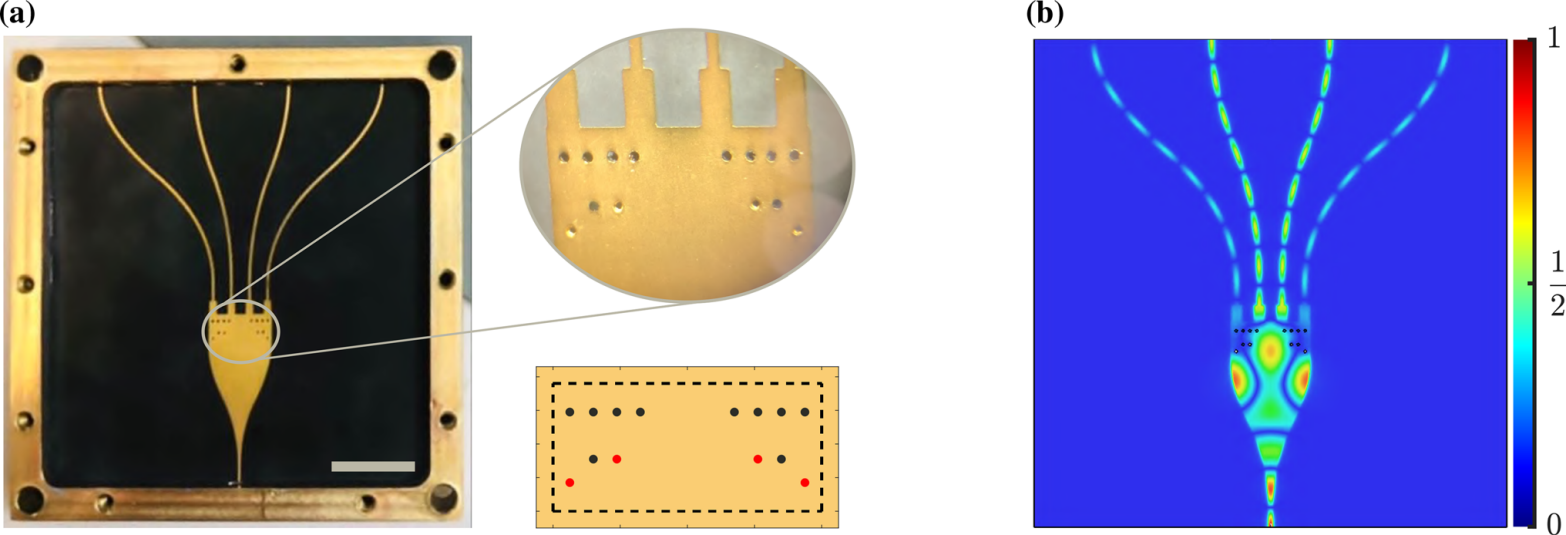
(**a**) Photograph of the fabricated power splitter embedded in a metal box. The gray bar in the photo corner corresponds to the length of 10 mm. A close-up view of the vias pattern, together with its schematical view, is shown. Black and red circles indicate lossy dielectric and metalized vias, respectively. (**b**) Normalized electric field distribution carried out by full-wave simulations.

**Figure 9:**
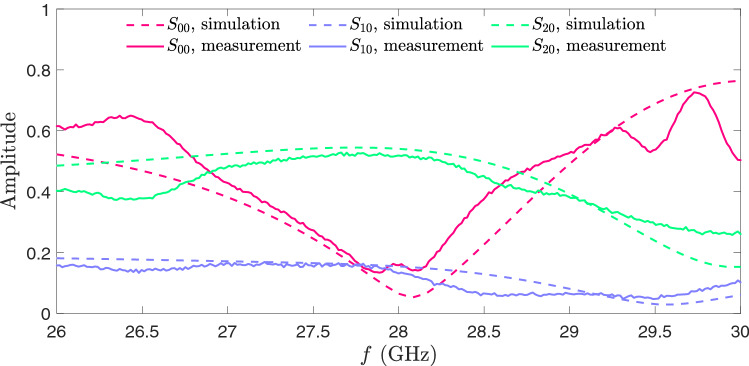
Reflection and transmission coefficients as functions of frequency carried out by simulations and measurements.

## Experimental results

We fabricated the proposed power splitter whose vias pattern is the combination of air and PEC material to verify our idea by demonstrating the power ratio of output ports experimentally, as shown in Fig. 8a. In the procedure fabrication, air vias are considered as non-metalized vias, and PEC vais are assumed as metalized vias, also gold plating has been used to reduce the effect of skin depth.

As already mentioned, based on Chebyshev coefficients, for a 4-element radiator providing 40 dB SLL, the amplitude ratios between the outer ports signal and the inner ports signal should be 0.37, i.e., $S_{10}/(S_{10} + S_{20}) =0.27$. The narrow gray shaded region shows this target’s satisfactory performance in Fig. 4e. Figure 9 denotes the spectral transmission from input port 0 to output port 1 and 2 ($S_{10}$, $S_{20}$) and spectral reflection of the input port ($S_{00}$) for the fabricated and simulated structure. For instance, we assumed that the return loss coefficient is lower than 15 dB and that the device’s loss is less than 0.9 dB over 28 GHZ as a center frequency. The measured results indicate that the proposed structure is a practical device for controlling SLL. Because we operate with a high-frequency setting that is sensitive to anything beyond fabrication tolerances and measurement restrictions, there are tiny discrepancies between simulation and fabrication results. The radiation field in Fig. 8b displays the electromagnetic behaviour of structure.

## Conclusion

We demonstrate a method by which microstrip power splitters with controllable output power are suggested. Through the design procedure, first, we investigate the dispersion of dielectric vias and the effects of their presence in the substrate. Then, we employ them, together with PEC vias, as inclusions in the multimode region of our device. PEC vias are very sparse, so the return loss of the power splitter remains acceptable. Using a neural network composed of two convolutional layers and one fully-connected layer, we are able to find an optimized pattern of hybrid perturbations, i.e., PEC and dielectric/air vias, for a target power division ratio. The training dataset dedicated to the neural network covers various cases, so almost diverse power division ratios for the device design are attainable with a satisfactory total transmittance. As a verification example, using the inverse network, we designed and fabricated a 4-output power divider at 28 GHz, by which Chebychef coefficients are realizable for SLL reduction purposes.

## Methods

### Numerical simulations

We utilized CST Studio Suite as a high-performance 3D EM analysis software package to design the power divider and generate the training data. We developed 1500 numerical simulations, where each experiment takes 1.63 min for a symmetrical setup with a desktop computer with a Core-i7 CPU with a 3.7 GHz clock speed and 32 GB RAM. The random vias position matrix of the power splitter was developed, exported for each case, and manipulated using MATLAB automatically.

### Inverse design method

We employed PyTorch, an open-source machine learning framework in Python, to build and test our inverse networks on a high-performance computer with a CPU board. Training parameters include a batch size of 128, epoch number of 10,000, and trained on 1500 data which takes 2440 s ($\sim 49$ min) to train the model. We analyze the design performance on arbitrary scenarios to assess the network’s capabilities.

## Data Availability

The authors confirm that the data supporting the finding of this study are available within the article and its Supplementary material. Raw data that support the findings of this study are available from the corresponding author, upon reasonable request.
